# Gut microbiome dysbiosis during COVID-19 is associated with increased risk for bacteremia and microbial translocation

**DOI:** 10.21203/rs.3.rs-726620/v1

**Published:** 2021-07-27

**Authors:** Mericien Venzon, Lucie Bernard-Raichon, Jon Klein, Jordan E. Axelrad, Grant A. Hussey, Alexis P. Sullivan, Arnau Casanovas-Massana, Maria G. Noval, Ana M. Valero-Jimenez, Juan Gago, Evan Wilder, Yale IMPACT Research Team, Lorna E. Thorpe, Dan R. Littman, Meike Dittmann, Kenneth A. Stapleford, Bo Shopsin, Victor J. Torres, Albert I. Ko, Akiko Iwasaki, Ken Cadwell, Jonas Schluter

**Affiliations:** 1 Vilcek Institute of Graduate Biomedical Sciences, New York University Grossman School of Medicine, New York, NY, USA; 2 Kimmel Center for Biology and Medicine at the Skirball Institute, New York University Grossman School of Medicine, New York, NY, USA; 3 Department of Immunobiology, Yale University School of Medicine, New Haven, CT, USA; 4 Division of Gastroenterology, Department of Medicine, New York University Grossman School of Medicine, New York, NY, USA; 5 Institute for Computational Medicine, New York University Grossman School of Medicine, New York, NY, USA; 6 Department of Epidemiology of Microbial Diseases, Yale School of Public Health, New Haven, CT, USA; 7 Department of Microbiology, New York University Grossman School of Medicine, New York, NY, USA; 8 Department of Population Health, New York University Grossman School of Medicine, New York, NY, USA; 9 Howard Hughes Medical Institute, Chevy Chase, MD; 10 Department of Medicine, Division of Infectious Diseases, New York University Grossman School of Medicine, New York, NY, USA; 11 IMPACT team members listed in the appendix

**Keywords:** microbes, COVID-19, secondary infectionsgut microbiome

## Abstract

The microbial populations in the gut microbiome have recently been associated with COVID-19 disease severity. However, a causal impact of the gut microbiome on COVID-19 patient health has not been established. Here we provide evidence that gut microbiome dysbiosis is associated with translocation of bacteria into the blood during COVID-19, causing life-threatening secondary infections. Antibiotics and other treatments during COVID-19 can potentially confound microbiome associations. We therefore first demonstrate that the gut microbiome is directly affected by SARS-CoV-2 infection in a dose-dependent manner in a mouse model, causally linking viral infection and gut microbiome dysbiosis. Comparison with stool samples collected from 101 COVID-19 patients at two different clinical sites also revealed substantial gut microbiome dysbiosis, paralleling our observations in the animal model. Specifically, we observed blooms of opportunistic pathogenic bacterial genera known to include antimicrobial-resistant species in hospitalized COVID-19 patients. Analysis of blood culture results testing for secondary microbial bloodstream infections with paired microbiome data obtained from these patients suggest that bacteria translocate from the gut into the systemic circulation of COVID-19 patients. These results are consistent with a direct role for gut microbiome dysbiosis in enabling dangerous secondary infections during COVID-19.

A better understanding of factors contributing to the pathology of coronavirus disease 2019 (COVID-19) is an urgent global priority. Infections by SARS-CoV-2 are frequently asymptomatic or mild in nature, but may also cause a broad range of severe and life-threatening symptoms. Previous reports have demonstrated that severe COVID-19 is frequently associated with specific inflammatory immune phenotypes, lymphopenia, and a generally disproportionate immune response leading to systemic organ failure^1,2^. Even in mild cases, gastrointestinal symptoms are reported frequently, and recent studies reported that COVID-19 patients lose commensal taxa of the gut microbiome during hospitalization^3,4^. Differences in gut bacterial populations relative to healthy controls were observed in all COVID-19 patients, but most strongly in patients who were treated with antibiotics during their hospitalization^4^. Most recently, COVID-19 patients treated with broad spectrum antibiotics at admission were shown to have increased susceptibility to multi-drug resistant infections and nearly double the mortality rate from septic shock^5,6^, and a recent meta-analysis found that over 14% of 3,338 COVID-19 patients acquired a secondary bacterial infection^7^. However, the causal direction of the relationship between disease symptoms and gut bacterial populations is not yet clear.

Complex gut microbiota ecosystems can prevent the invasion of potentially pathogenic bacteria^8,9^. Conversely, when the gut microbiota incurs damage, such as through antibiotics treatment, competitive exclusion of pathogens is weakened^10^ and potentially dangerous blooms of antibiotic resistant bacterial strains can occur ^11,12^. In immunocompromised cancer patients, blooms of Enterococcaceae and Gram-negative proteobacteria can lead to gut dominations by few or single species^13–16^. Gut domination events are dangerous to these patients because antibiotic resistant bacteria may translocate from the gut into the blood stream. Consequently, enterococcal dominations have been associated with 9-fold increased risk of bloodstream infections (BSIs) with vancomycin-resistant Enterococcus (VRE), and domination by Gram-negative proteobacteria with 5-fold increased risk of Gram-negative rod BSIs^13^. Bacterial co-infection can also cause life-threatening complications in patients with severe viral infections^6,17^; therefore, antibacterial agents were administered empirically to nearly all critically ill suspected COVID-19 patients since the incidence of bacterial superinfection was unknown early during the pandemic^4,18^. However, it is now known that nosocomial infection during prolonged hospitalization is the primary threat to patients with COVID-19^19^, rather than bacterial co-infection upon hospital admission^7,20–22^. Evidence from immunocompromised cancer patients suggests that indiscriminate administration of broad-spectrum antibiotics may, counter-intuitively, increase nosocomial BSI rates by causing gut dominations of resistant microbes that can translocate into the blood^13,23^. Thus, empiric antimicrobial use, i.e. without direct evidence for a bacterial infection, in patients with severe COVID-19 may be especially pernicious because it may select for antimicrobial resistance and could promote gut translocation-associated BSI.

The role of the gut microbiome in respiratory viral infections in general^24^, and in COVID-19 patients in particular, is only beginning to be understood. Animal models of influenza virus infection have uncovered mechanisms by which the microbiome influences antiviral immunity^25–27^, and in turn, the viral infection was shown to disrupt the intestinal barrier of mice by damaging the gut microbiota^28,29^. Hence, we hypothesized that gut dysbiosis during COVID-19 may be associated with BSIs. To test this, we first determined whether SARS-CoV-2 infection could directly cause gut dysbiosis independently of hospitalization and treatment. Daily changes in fecal bacterial populations were monitored following intranasal inoculation of transgenic mice expressing human *ACE2* driven by the *cytokeratin-18* promoter (*K18-ACE2tg* mice) with either a high dose (HD,10^4^PFU) or low dose (LD, 10PFU) of SARS-CoV-2 (Figure 1a). Although disease was not as evident in LD mice, we confirmed the presence of infectious virus in the lung by plaque assay at sacrifice (Supplementary Figure S1). Among the HD mice, we observed significant microbiome changes (Figure 1b), with a repeatedly observed community trajectory corresponding to a loss in relative abundances of obligate anaerobe species such as members of the Clostridiales order (Figure 1c), concurrent with an expansion of Verrucomicrobiales (Figure 1a,c). During this shift in the microbiome, α-diversity in the gut bacterial ecosystem was decreasing, a trend also observed in the LD mice, albeit to a lesser extent, but not in the control mice (Figure 1b). After less than one week of viral infection, α-diversity was reduced in infected mice (Figure 1b, 95%HDI<0 Bayesian estimation of differences in group means BEST, methods). Alongside the progressive increase in microbiota compositional dysbiosis, we also observed systemic signs of severe infection, including weight loss (Supplementary Figure S2), as well as ruffled fur, heavy breathing, reduced activity and hunched posture (Supplementary Table S1). These results demonstrate that SARS-CoV2 infection directly causes gut microbiome dysbiosis in a mouse model.

We next profiled the bacterial composition of the fecal microbiome in 138 samples (Figure 2a) obtained from SARS-CoV-2 infected patients treated at NYU Langone Health (NYU, 73 samples, Supplementary Table S2) and Yale New Haven Hospital (YALE, 65 samples, Supplementary Table S3). Analysis of metagenomic data obtained from sequencing of the 16S rRNA genes revealed a wide range of bacterial community diversities, as measured by the inverse Simpson index, in samples from both centers (NYU: [1.0, 32.2], YALE: [1.5, 29.3], Figure 2b); on average, samples from NYU were less diverse (−2.5, p<0.01, Figure 2c). However, the composition in samples between the two centers did not show systematic compositional differences (Figure 2d,e,f). On average, in both centers, members of the phyla Firmicutes and Bacteroidetes represented the most abundant bacteria, followed by Proteobacteria (Figure 2d). The wide range of bacterial diversities was reflected in the high variability of bacterial compositions across samples (Figure 2e,f). In samples from both centers, microbiome dominations, defined as a community where a single genus reached more than 50% of the population, were observed frequently (NYU: 21 samples, YALE: 12 samples), representing states of severe microbiome injury in COVID-19 patients (Figure 2g).

In agreement with a recent study associating gut microbial compositions with disease severity^4^, we found that samples from patients who were treated in the ICU had reduced bacterial diversity (Supplementary Figure S3). In 22 cases, gastrointestinal symptoms were recorded, but only two of those patients required ICU treatment and corresponding stool samples had higher average diversity than other samples (Supplementary Figure S3). Strikingly, however, samples associated with a BSI had strongly reduced bacterial α-diversities (mean difference: −7.7, CI_BEST_[−10.2, −5.2], Supplementary Figure S3).

The lower diversity associated with samples from 21 patients with BSIs led us to investigate their bacterial taxon compositions and the potential that gut dysbiosis was associated with BSI events. All BSI patients had received antibiotic treatments during hospitalization, which could exacerbate COVID-19 induced shifts in microbiota populations^11,12,15^, but may indeed be administered in response to a suspected or confirmed BSI. However, we noted that most BSI patients (80%) also received antibiotics prior to their BSI. Principal coordinate analysis of all stool samples indicated that the BSI-associated samples spanned a broad range of compositions (Figure 3a). To identify bacterial abundance patterns that consistently distinguished BSI from non-BSI-associated samples, we next performed a Bayesian logistic regression. This analysis estimated the association of the 10 most abundant bacterial genera with BSI cases, i.e. it identified enrichment or depletion of bacterial genera in BSI associated samples (Figure 3b). This analysis revealed that the genus *Faecalibacterium* was negatively associated with BSI (OR: −1.49, CI:[−2.82, −0.18]). *Faecalibacterium* is an immunosupportive Clostridiales genus that is a prominent member of the human gut microbiome^30–32^, and its reduction is associated with disruption to intestinal barrier function^33,34^, perhaps via ecological network effects^34^.

To evaluate the effect size of the association between *Faecalibacterium* and BSIs, we performed a posterior predictive check. Using the average genus composition found across all samples, we first computed the distribution of predicted BSI risks (Figure 3c), and compared this risk distribution with a hypothetical bacterial composition which increased *Faecalibacterium* by 10% points. The predicted risk distributions associated with these two compositions differed strongly (mean difference 26%, CI: [−9%, 67%], Figure 3c). Domination states of the microbiome increase the risk for BSIs in immunocompromised cancer patients ^13^; such dominations imply high relative abundances of single taxa, and therefore a low diversity. Consistent with this, *Faecalibacterium* abundance was positively correlated with diversity (R: 0.55, p<10^−10^, Figure 3d) in our data set and as reported previously ^30^.

We therefore next investigated a direct association between the bacteria populating the gut microbiome and the organisms identified in the blood of patients. Visualizing the bacterial composition in stool samples from patients alongside the BSI microorganism (Supplementary Figure S4, Figure 3e) suggested a correspondence with the respective taxa identified in the blood: high abundances of the BSI-causing microbes were found in corresponding stool samples (Figure 3e). To analyze this, we first assigned stool samples associated with each BSI event into 5 categories defined by the taxonomic order of the causative bacterial organisms, as well as one singleton group of a fungal infection case as a sixth category; stool samples from uninfected patients were assigned a seventh, “uninfected” category. For samples of each BSI category, we first calculated their median abundances of bacterial predictors in the stool. We then ranked these stool taxon median abundances across BSI categories. As expected from the visualization of sample compositions (Figure 3e), we found that BSI category sample sets were generally enriched in their respective taxa in the stool. For example, samples associated with *Klebsiella*, *Escherichia* or *Serratia* BSIs (Enbct category, Figure 3f) had the highest rank of Enterobacterales abundances across the BSI category sample sets (Figure 3f). We tested this observation statistically using the log_10_-relative bacterial abundances in stool samples as independent predictors of identified BSI pathogens, i.e. the BSI category, in a Bayesian categorical regression model where the uninfected class was used as a pivot (see methods). In addition to taxon abundances, the model included the bacterial α-diversity as a predictor. As expected, a strong statistical association between diversity and BSIs in general was detected (Supplementary Figure S5). The rank analysis had suggested that Staphylococcales are not enriched in BSIs by *Staphylococcus* (Figure 3f); this was supported by the Bayesian model which showed that log_10_-abundances of Staphylococcales in the stool were not detectably predictive of a *Staphylococcus* BSI (Figure 3g). By contrast, our analysis demonstrated that the bacterial abundances of all other BSI-causing organisms in the stool were predictive of corresponding BSIs.

Collectively, these results reveal an unappreciated link between SARS-CoV-2 infection, gut microbiome dysbiosis, and a major complication of COVID-19, BSIs. The loss of diversity and immunosupportive *Faecalibacterium* in patients with BSIs mirrored a similar loss of diversity and Clostridiales in the mice receiving high doses of SARS-CoV-2, suggesting that this virus causally affects the microbiome, either through direct infection^35–39^ or through a systemic inflammatory response ^2,4^. However, the dysbiosis in patients with COVID-19 exceeded the microbiota shifts observed in the mouse experiments, including microbiome dominations by single taxa, which was not seen in the mouse experiments. It is possible that in our experiment, mice were sacrificed before perturbations to the gut microbial populations reached a maximum. However, it is also plausible that the frequently administered antibiotic treatments that hospitalized COVID-19 patients receive exacerbated SARS-CoV-2 induced microbiome perturbations. Additionally, unlike the controlled environment experienced by laboratory mice, hospitalized patients are uniquely exposed to antimicrobial-resistant infectious agents present on surfaces and shed by other patients. Indeed, domination events where the gut is populated by only a few taxa have been described in hospitalized, immunocompromised cancer patients treated with broad spectrum antibiotics^15^. We frequently observed such dominations in our COVID-19 cohorts treated at two hospitals.

Our observation that the type of bacteria that entered the bloodstream was disproportionately enriched in the associated stool samples is a well characterized phenomenon in cancer patients^13^, especially during chemotherapy induced leukocytopenia when patients are severely immunocompromised^11,30^. COVID-19 patients are also immunocompromised and frequently incur lymphopenia, rendering them susceptible to secondary infections^40^. Our data suggests dynamics in COVID-19 patients may be similar to those observed in cancer patients: BSI-causing organisms may translocate from the gut into the blood, potentially due to loss of gut barrier integrity, through virus-induced tissue damage rather than chemotherapy. Consistent with this possibility, soluble immune mediators such as TNFa and interferons produced during viral infections, including SARS-CoV-2, damage the intestinal epithelium to disrupt the gut barrier, especially when the inflammatory response is sustained as observed in patient with severe COVID-19^41–43^.

One limitation of our data is temporal ordering of samples. Occasionally stool samples were collected after observation of BSI, and this mismatch in temporal ordering is counter intuitive for gut-to-blood translocation and a causal interpretation of our associations. However, the reverse direction, that blood infection populates and changes the gut community, is unlikely for the organisms identified in the blood, and if our associations were not causal, we would expect no match between BSI organisms and stool compositions.

Taken together, our findings support a scenario in which gut-to-blood translocation of microorganisms following microbiome dysbiosis, a known issue for chronic conditions such as cancer, leads to dangerous BSIs during COVID-19. We suggest that investigating the underlying mechanism behind our observations will inform the judicious application of antibiotics and immunosuppressives in patients with respiratory viral infections and increase our resilience to pandemics.

## Materials and Methods

### Bioethics statement

The collection of COVID-19 human biospecimens for research has been approved by the NYUSOM Institutional Review Board under il8–01121 Inflammatory Bowel Disease and Enteric Infection at NYU Langone Health. The data presented in this study were also approved by Yale Human Research Protection Program Institutional Review Boards (FWA00002571, protocol ID 2000027690). Informed consent was obtained from all enrolled patients.

### Mouse experiments

#### Cells & virus

Vero E6 (CRL-1586; American Type Culture Collection) were cultured Dulbecco’s Modified Eagle’s Medium (DMEM,Corning) supplemented with 10% fetal bovine serum (FBS, Atlanta Biologics) and 1% nonessential amino acids (NEAA,Corning). SARS-CoV-2, isolate USA-WA1/2020 19 (BEI resources #NR52281), a gift from Dr. Mark Mulligan at the NYU Langone Vaccine Center was amplified once in Vero E6cells. All experiments with SARS-CoV-2 were conducted in the NYU Grossman School of Medicine ABSL3 facility by personnel equipped with powered air-purifying respirators.

#### Mice

Heterozygous K18-hACE2 C57BL/6J mice (strain: 2B6.Cg-Tg(K18-ACE2)2Prlmn/J) were obtained from The Jackson Laboratory. Animals were housed in groups and fed standard chow diets. All animal studies were performed according to protocols approved by the NYU School of Medicine Institutional Animal Care and Use Committee (IACUC n°170209). 24-week-old K18-hACE2 males were administered either 10PFU SARS-CoV-2 (low dose, LD), 10^4^PFU SARS-CoV-2 (high dose, HD) diluted in 50μL PBS (Corning) or 50μL PBS (non-infected, CTRL) via intranasal administration under xylazine-ketamine anesthesia (AnaSedR AKORN Animal Health, KetathesiaTM Henry Schein Inc). Viral titer in the inoculum was verified by plaque assay in Vero E6 cells. Following infection, mice were monitored daily for weight loss and signs of disease. Stool samples were collected and stored at −80°C.

#### Measurement of viral load by plaque assay

Six or seven days after infection, mice were sacrificed. For some mice lungs were collected in Eppendorf tubes containing 500μl of PBS and a 5mm stainless steel bead (Qiagen) and homogenized using with the Qiagen TissueLyser II. Homogenates were cleared for 5 min at 5,000 × g, and viral supernatant was frozen at −80°C for titration through plaque assay. In brief, Vero E6 cells were seeded at a density of 2.2 * 10^5^ cells per well in flat-bottom 24-well tissue culture plates. The following day, media was removed and replaced with 100μL of tenfold serial dilutions of the virus stock, diluted in infection medium. Plates were incubated for 1h at 37°C. Following incubation, cells were overlaid with 0.8% agarose in DMEM containing 2% FBS and incubated at 37°C for 72hrs. Cells were then fixed with formalin buffered 10% (Fisher Chemical) for 1h. Agarose plugs were then removed and cells were stained for 20 min with crystal violet and then washed with tap water.

### Human study population and data collection

This study involved 101 patients with laboratory-confirmed SARS-CoV-2 infection. SARS-CoV-2 infection was confirmed by a positive result of real-time reverse transcriptase-polymerase chain reaction assay on a nasopharyngeal swab. 64 patients were seen at NYU Langone Health, New York, for routine medical procedures, outpatient care, or admitted through the Emergency Department at NYU Langone Health’s Tisch Hospital, New York City, between January 29, 2020 – July 2, 2020 and were followed until discharge. In order to be eligible for inclusion in the study, stool specimens needed to be from individuals >18 years of age. Data including demographic information, clinical outcomes, and laboratory results were extracted from the electronic medical records in the NYU Langone Health clinical management system. Blood and stool samples were collected by hospital staff. OmnigeneGut kits were used on collected stool. In parallel, 37 patients were admitted to YNHH with COVID-19 between 18 March 2020 and 27 May 2020 as part of the YALE IMPACT cohort described at length elsewhere^2^. Briefly, participants were enrolled after providing informed consent and paired blood and stool samples were collected longitudinally where feasible for duration of hospital admission. No statistical methods were used to predetermine sample size for this cohort. Demographic information of patients was aggregated through a systematic and retrospective review of the EHR and was used to construct Supplementary Table 3. Symptom onset and aetiology were recorded through standardized interviews with patients or patient surrogates upon enrolment in our study, or alternatively through manual EHR review if no interview was possible owing to clinical status at enrolment. The clinical data were collected using EPIC EHR and REDCap 9.3.6 software. At the time of sample acquisition and processing, investigators were blinded to patient clinical status.

### DNA extraction and bacterial 16S rRNA sequencing

For bacterial DNA extraction 700μL of SL1 lysis buffer (NucleoSpin Soil kit, Macherey-Nagel) was added to the stool samples and tubes were heated at 95°C for 2h to inactivate SARS-CoV-2. Samples were then homogenized using the FastPrep-24TM instrument (MP Biomedicals) and extraction was pursued using the NucleoSpin Soil kit according to the manufacturer’s instructions. DNA concentration was assessed using a NanoDrop spectrophotometer. Samples with too low DNA concentration were excluded. DNA from human samples was extracted with PowerSoil Pro (Qiagen) on the QiaCube HT (Qiagen), using Powerbead Pro (Qiagen) plates with 0.5mm and 0.1mm ceramic beads. For mouse samples, the variable region 4 (V4) of the 16S rRNA gene was amplified by PCR using primers containing adapters for MiSeq sequencing and single-index barcodes. All PCR products were analyzed with the Agilent TapeStation for quality control and then pooled equimolar and sequenced directly in the Illumina MiSeq platform using the 2×250 bp protocol. Human samples were prepared with a protocol derived from ^44^, using KAPA HiFi Polymerase to amplify the V4 region of the 16S rRNA gene. Libraries were sequenced on an Illumina MiSeq using paired-end 2×250 reads and the MiSeq Reagent Kitv2.

### Bioinformatic processing and taxonomic assignment

Amplicon sequence variants (ASVs) were generated via dada2 v1.16.0 using post-QC FASTQ files. Within the workflow, the paired FASTQ reads were trimmed, and then filtered to remove reads containing Ns, or with maximum expected errors >= 2. The dada2 learn error rate model was used to estimate the error profile prior to using the core dada2 algorithm for inferring the sample composition. Forward and reverse reads were merged by overlapping sequence, and chimeras were removed before taxonomic assignment. ASV taxonomy was assigned up to genus level using the SILVAv.138 database with the method described in ^45^ and a minimum boostrapping support of 50%. Species-level taxonomy was assigned to ASVs only with 100% identity and unambiguous matching to the reference.

### Compositional analyses

#### α-Diversity

We calculated the inverse Simpson (*IVS*) index from relative ASV abundances (*p*) with *N* ASVs in a given sample, $IVS=\frac{1}{\sum_{i}^{N}p_{i}^{2}}$.

#### Principal Coordinate Analyses

Bray-Curtis distances were calculated from the filtered ASV table using QIIME 1.9.1 and principal components of the resulting distance matrix were calculated using the scikit-learn package for the Python programming language, used to embed sample compositions in the first two principal coordinates (see published code for the implementation in the Python programming language).

#### Average compositions and manipulation of compositions

To describe the average composition of a set of samples we calculated the central tendency of a compositional sample ^46^. For counter factual statistical analyses that require changes to a composition, e.g. an increase in a specific taxon, we deployed the perturbation operation (⊕), which is the compositional analogue to addition in Euclidean space46. A sample *x* containing the original relative taxon abundances is perturbed by a vector *y*,

$$y:\;x⊕y=\left[\frac{x_{1}y_{1}}{\sum_{i=1}^{D}x_{i}y_{i}},\frac{x_{2}y_{2}}{\sum_{i=1}^{D}x_{i}y_{i}},\ldots ,\frac{x_{D}y_{D}}{\sum_{i=1}^{D}x_{i}y_{i}}\right]\forall x,y\in S^{D}$$

where *S*^*D*^ represents the D-part simplex.

### Statistical analyses

Computer code alongside processed data tables are made available and can be used to reproduce the statistical analysis and regenerate the figures (**Supplementary File 1**).

#### Bayesian t-test

To compare diversity measurements between different sample groups, e.g. different clinical status, we performed a Bayesian estimation of group differences (BEST, ^47^) , implemented using the pymc3 package for the Python programming language; with priors (~) and deterministic calculations (=) to assess differences in estimated group means as follows:

$${\text{g}}_{1}∼\mathrm{Normal}(\text{μ}=15,\text{σ}=15)$$

$${\text{g}}_{2}∼\mathrm{Normal}(\text{μ}=15,\text{σ}=15)$$

$${\text{σ}}_{\text{g}1}∼\text{Uniform}(\text{low}=1\text{e}-4,\text{high}=30)$$

$${\text{σ}}_{\text{g}2}∼\text{Uniform}(\text{low}=1\text{e}-4,\text{high}=30)$$

ν∼Exponential(1/15)+1

$${\text{λ}}_{1}={{\text{σ}}_{\text{g}1}}^{-2}$$

$${\text{λ}}_{2}={{\text{σ}}_{\text{g}2}}^{-2}$$

$$\text{G1}∼\text{StudentT}\left(\text{nu}=\text{ν},\text{mu}={\text{g}}_{1},\text{lam}={\text{λ}}_{1}\right)$$

$$\text{G2}∼\text{StudentT}\left(\text{nu}=\text{ν},\text{mu}={\text{g}}_{2},\text{lam}={\text{λ}}_{2}\right)$$

Δ=G1−G2

Bayesian inference was performed using “No U-turn sampling”^48^. Highest density intervals (HDI) of the posterior estimation of group differences (Δ) were used to determine statistical certainty (***: 99% HDI >0 or <0, **: 95%HDI, *:90% HDI). The BEST code is provided in the Supplementary and implemented following the pymc3 documentation (**Supplementary File 1**).

#### Bayesian logistic regression

We performed a Bayesian logistic regression to distinguish compositional differences between infection-associated samples and samples from patients without secondary infections. We modeled the infection state of patient sample *i*, *yi* with a Binomial likelihood:

$${\text{y}}_{\text{i}}∼\text{Binomial}(\text{n}=1,\text{p}=\text{p})$$

$$\text{p}=\text{inverse}\;\text{logistic}\left(\text{α}+{\text{X}}_{\text{i}}\text{β}\right)$$

α∼Normal(μ=0,σ=1)

β∼Normal(μ=0,σ=1)

Where prior distributions are indicated by ~; α is the intercept of the generalized linear model, β is the coefficient vector for the log_10_-relative taxon abundances *X*_*i*_ in sample *i*.

#### Bayesian categorical regression

To interrogate a correspondence between the taxon abundances in stool samples and the microorganisms causing BSIs, we performed a Bayesian categorical regression. Briefly, we chose to investigate an association between stool taxon abundances (independent predictor variable) and the microbe identified in the blood (categorical outcome variable with seven unordered values) using a multiclass regression (categorical regression). We estimated for each sample a probability of being associated with one of the 6 BSI types (i.e. BSI by: Bacteroidaceae, Enterobacteriaceae, Lactobacillaceae, Pseudomonadaceae, Staphylococcaceae, Saccharomycetaceae), and we used a seventh class, uninfected, as a pivot. This means we are estimating a seven component simplical vector (*s*) containing the probabilities of a sample to be associated with one of the seven categories (6 BSI types and uninfected). For each category, we set up a linear model (s_g_, where _g_ indicates the category). Each linear model includes log_10_-relative stool taxon abundances of the taxa corresponding to the BSI category. Furthermore, we had shown that alpha diversity (IVS) was globally associated with BSI; thus, diversity was a predictor in each linear model. We set up a model using varying intercept and varying slope terms such that the linear models used partially pooled coefficients for baseline risks (β[1]) and slopes corresponding to the stool sample predictors (β[2]). The multiclass probabilities in *s* were then obtained by applying the softmax function. The following model and priors were used:

$${\text{y}}_{\text{i}}∼\text{Categorical}(\text{p}=\text{p})$$

p=softmax(s)

z=(σp∗Lp)∗zp

$${\text{s}}_{\text{Bacteroidaceae}}=\text{β}[1]+\text{z}[1,1]+(\text{β}[2]+\text{z}[2,1])*{\text{X}}_{\text{Bacteroidaceae}}+{\text{β}}_{\text{diversity}}*\text{IVS}$$

$${\text{s}}_{\text{Enterobacteriaceae}}=\text{β}[1]+\text{Z}[1,2]+(\text{β}[2]+\text{z}[2,2])*{\text{X}}_{\text{Enterobacteriaceae}}+{\text{β}}_{\text{diversity}}*\text{IVS}$$

$${\text{s}}_{\text{Lactobacillaceae}}=\text{β}[1]+\text{z}[1,3]+(\text{β}[2]+\text{z}[2,3])*{\text{X}}_{\text{Lactobacillaceae}}+{\text{β}}_{\text{diversity}}*\text{IVS}$$

$${\text{s}}_{\text{Pseudomonadaceae}}=\text{β}[1]+\text{z}[1,4]+(\text{β}[2]+\text{z}[2,4])*{\text{X}}_{\text{Pseudomonadaceae}}+{\text{β}}_{\text{diversity}}*\text{IVS}$$

$${\text{s}}_{\text{Staphylococcaceae}}={\text{α}}_{\text{Staphylococcaceae}}+{\text{β}}_{\text{Staphylococcaceae}}*{\text{X}}_{\text{Staphylococcaceae}}+{\text{β}}_{\text{diversity}}*\text{IVS}$$

$${\text{s}}_{\text{Saccharomycetaceae}}={\text{α}}_{\text{Saccharomycetaceae}}+{\text{β}}_{\text{diversity}}*\text{IVS}$$

$${\text{s}}_{\text{uninfected}}=0$$

β∼Normal(μ=0,σ=1)

σ∼Exponential(λ=1)

σp∼Exponential(λ=1)

Lp∼LKJ_corr_choleski(2)

$${\text{α}}_{\text{Staphylococcaceae}}∼\text{Normal}(\text{μ}=0,\text{σ}=1)$$

$${\text{β}}_{\text{Staphylococcaceae}}∼\text{Normal}(\text{μ}=0,\text{σ}=1)$$

$${\text{β}}_{\text{diversity}}∼\text{Normal}(\text{μ}=0,\text{σ}=1)$$

$${\text{α}}_{\text{Saccharomycetaceae}}∼\text{Normal}(\text{μ}=0,\text{σ}=2)$$

zp∼Normal(μ=0,σ=1)

Where p corresponds to the probabilities of each category, β is a two-component variable for the intercept and slope terms, representing the global baseline probability of bacterial infections with Bacteroidaceae, Enterobacteriaceae, Lactobacillaceae, and Pseudomonadaceae as well as the global slope coefficient for the effect of stool log_10_-relative abundances, which are partially pooling information across bacterial infection categories. To achieve partial pooling and account for correlations between varying intercepts and slopes (*z*), we jointly inferred the Choleski-factorized covariance matrix (σp, Lp, zp), using the Lewandowski-Kurowicka-Joe (LKJ) distribution as a prior (LKJ_corr_choleski). β_diversity_ is the coefficient for the effect of the *IVS* diversity. Of note, 16S rRNA sequencing does not provide abundances for the fungal infection by Saccharomycetaceae; therefore, we used only a baseline risk for this infection type (α_Saccharomycetaceae_) and IVS as predictors. To ensure equal prior probabilities for this category relative to the other categories, which have an additional predictor term and thus wider prior probabilities, we compensated the otherwise reduced prior uncertainty by widening the prior for α_Saccharomycetaceae_. Also, we assumed that infections by Staphylococcaceae could sometimes include contaminations from the skin of the patient or staff; therefore, we did not pool estimates for BSIs by Staphylococcaceae with other coefficients. The model was implemented in the STAN programming language and compiled using cmdstan. Code, the compiled STAN model, R notebooks to obtain and process the posterior chains, and data tables are provided in the supplement (**Supplementary File 2**).

### Data Availability

All data is made available as Supplementary. We provide code and data to reproduce the main analyses in the form of jupyter notebooks and R notebooks, alongside processed “tidy” data tables, compiled STAN programs and code to regenerate the figures. The raw sequencing data have been deposited on the Sequencing Reads Archive (SRA), and SRA accession numbers are available for two bioprojects corresponding to the mouse sequencing data (**Supplementary File 3**) and the human stool samples (**Supplementary File 4**).

## Supplementary Material

Supplement 1

## Figures and Tables

**Figure 1.**
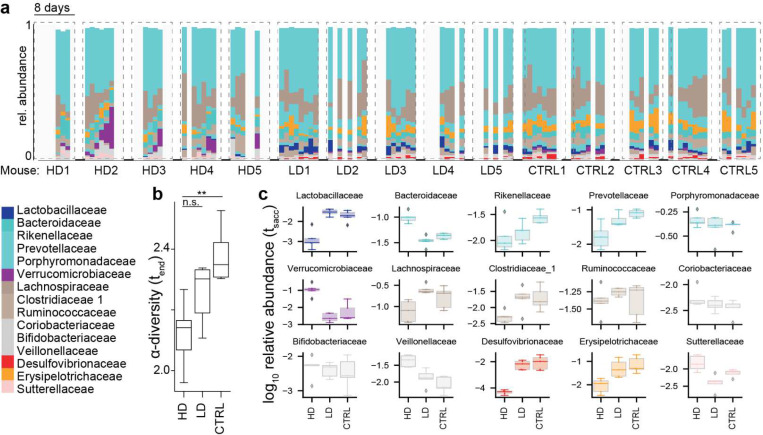
SARS-CoV-2 infection causes gut microbiome alterations in mice. **a** Timelines of fecal microbiota composition measured by 16S rRNA gene sequencing in mice infected with high (HD, 10^4^PFU), or low doses (LD, 10PFU) and in uninfected control mice (CTRL); time of infection=Day 1. Bars represent the composition of the 30 most abundant bacterial families per sample, blocks of samples correspond to an individual mouse’s time course. **b** *α*-diversity (Shannon) in the final samples per infection group; **: HDI95<0 BEST. **c** *log*_10_-relative family abundances at the final time point. heavy breathing, reduced activity and hunched posture (Supplementary Table S1).

**Figure 2.**
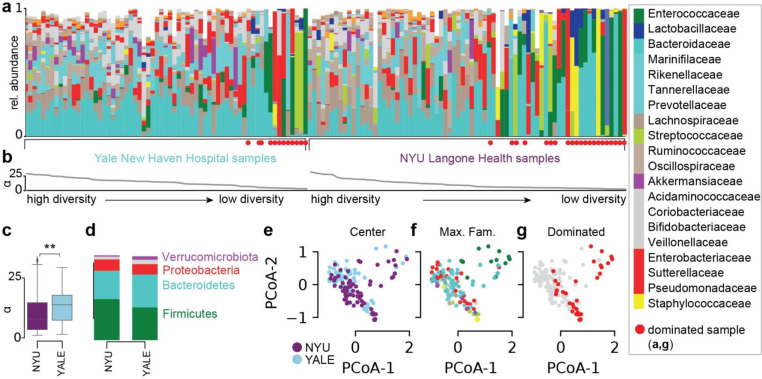
Gut microbiome bacterial compositions during COVID-19 in patients from NYU Langone Health and Yale New Haven Hospital. **a** Bacterial family composition in stool samples identified by 16S rRNA gene sequencing; bars represent the relative abundances of bacterial families; red circles indicate samples with single taxa >50%. Samples are sorted by the bacterial *α*-diversity (inverse Simpson index, **b**). **c** *α*-diversity in samples from NYU Langone Health and Yale New Haven Hospital. **d** Average phylum level composition per center. **e-g** Principal coordinate plots of all samples shown in **a**, labeled by center (**e**), most abundant bacterial family (**f**) and domination status of the sample (**g**).

**Figure 3.**
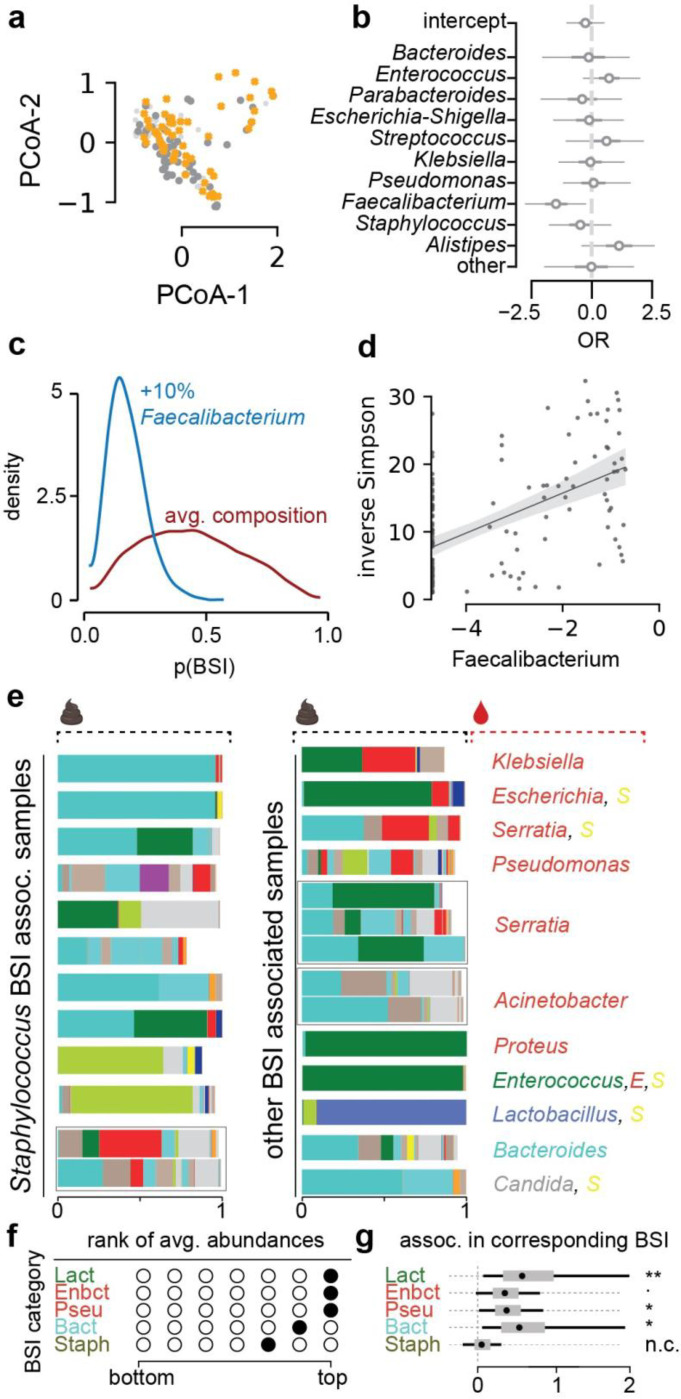
Microbiome composition is associated with secondary bloodstream infections. **a** Principal coordinate plot of all samples, BSI associated samples in orange. **b** Posterior coefficient estimates from a Bayesian logistic regression regressing log_10_ relative abundances of the top 10 most abundant bacterial genera on BSI status. **c** Posterior prediction of BSI risk based on bacterial composition contrasting the predicted risk of the average composition across all samples (red) with the risk estimated for the same composition changed such that *Faecalibacterium* was increased by 10% (blue). **d** Log_10_ relative abundances of *Faecalibacterium* correlated with *α*-diversity, shaded region: 95%CI. **e** Sample compositions with BSIs indicated; left: *Staphylococcus* BSI associated samples; right: other BSI associated samples, the BSI causing microbial genus annotated in colors corresponding to the colors in stool microbiome compositions. **f** Rank analysis of abundance patterns in stool samples from different BSI categories; a filled circle indicates the calculated rank of the focal BSI category (row) in terms of the corresponding taxon stool abundance relative to samples from other BSI categories (only 5 out of 7 BSI categories are shown because fungal BSIs and the uninfected category have no corresponding bacterial stool abundances). **g** Posterior coefficients of the statistical association between bacterial order log_10_ relative abundances of BSI causing bacteria and BSI events from a hierarchical Bayesian categorical regression; **: 95%HDI>0, *: 90%HDI>0, .:85% HDI>0.

## Yale IMPACT Team

Abeer Obaid, Alice Lu-Culligan, Allison Nelson, Anderson Brito, Angela Nunez, Anjelica Martin, Annie Watkins, Bertie Geng, Chaney Kalinich, Christina Harden, Codruta Todeasa, Cole Jensen, Daniel Kim, David McDonald, Denise Shepard, Edward Courchaine, Elizabeth B. White, Eric Song, Erin Silva, Eriko Kudo, Giuseppe DeIuliis, Harold Rahming, Hong-Jai Park, Irene Matos, Jessica Nouws, Jordan Valdez, Joseph Fauver, Joseph Lim, Kadi-Ann Rose, Kelly Anastasio, Kristina Brower, Laura Glick, Lokesh Sharma, Lorenzo Sewanan, Lynda Knaggs, Maksym Minasyan, Maria Batsu, Mary Petrone, Maxine Kuang, Maura Nakahata, Melissa Campbell, Melissa Linehan, Michael H. Askenase, Michael Simonov, Mikhail Smolgovsky, Nicole Sonnert, Nida Naushad, Pavithra Vijayakumar, Rick Martinello, Rupak Datta, Ryan Handoko, Santos Bermejo, Sarah Prophet, Sean Bickerton, Sofia Velazquez, Tara Alpert, Tyler Rice, William Khoury-Hanold, Xiaohua Peng, Yexin Yang, Yiyun Cao & Yvette Strong
