# Supplementary material for: Gut microbiome dysbiosis during COVID-19 is associated with increased risk for bacteremia and microbial translocation
Source: Res Sq. 2021 Jul 27:rs.3.rs-726620. Preprint. [Version 1] doi: 10.21203/rs.3.rs-726620/v1 (PMC8328072; doi:10.21203/rs.3.rs-726620/v1)
Supplement: Supplement 1 [file bdecfc8ee2db4b7235d5c601.pdf]

Supporting Information

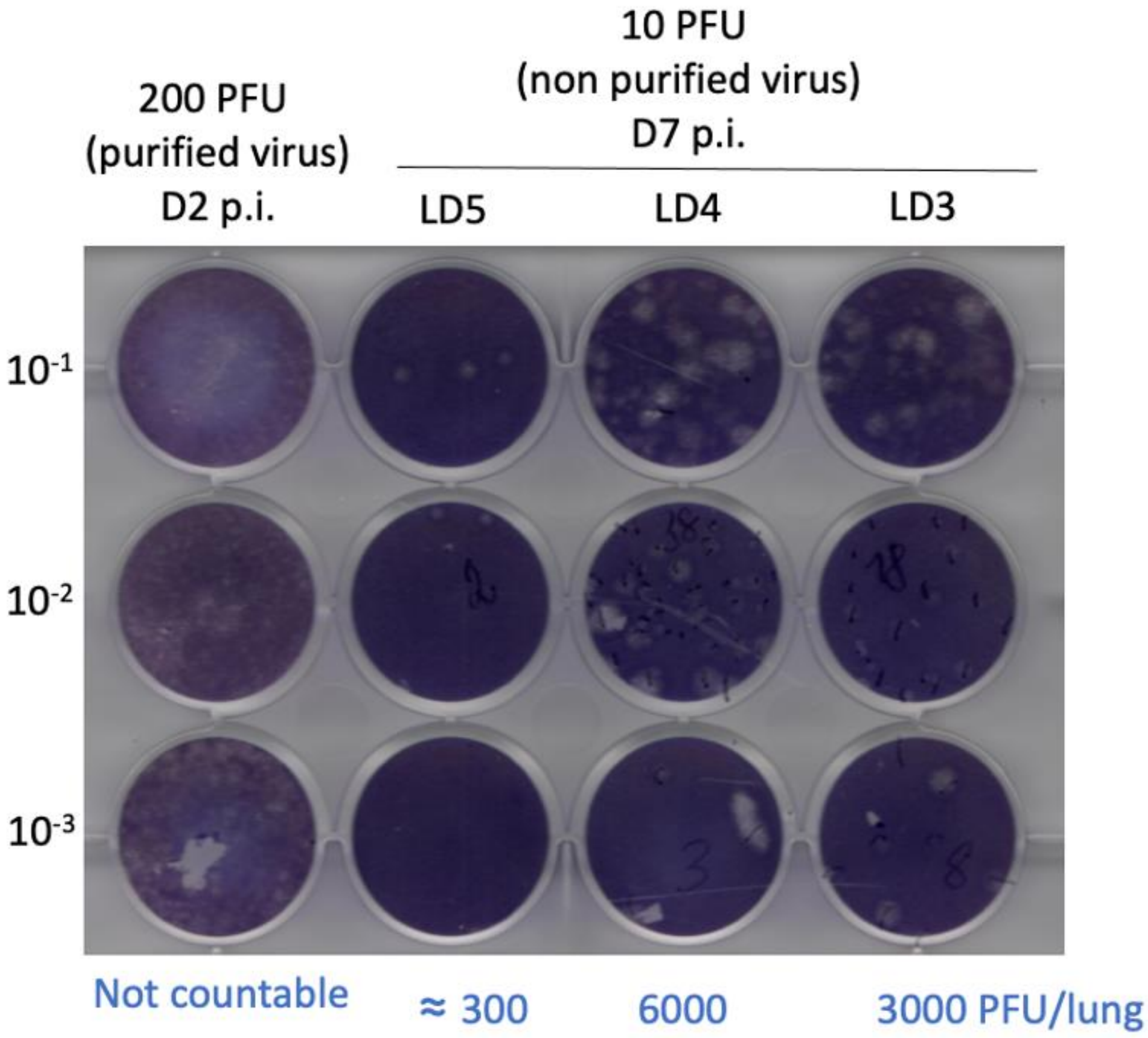

Supplementary Figure S1. Plaque assay; SARS-CoV2 replicates in the lungs of *K18-ACE2tg* mice infected with 10 PFU. Lungs from mice LD3, LD4, LD5 were harvested 7 days post-infection and homogenized in 500uL PBS. A lung collected 2 days post-infection from a mouse infected with 200 PFU in another experiment was used as positive control (left). Viral load in the homogenates was determined by plaque assay by incubation for 1h of 1/10 (top), 1/100 (middle) or 1/1000 (bottom) dilutions of the homogenates on Vero E6 cells. After 1h incubation, cells were overlayed with agarose containing medium. 72h post infection, the cells were fixed and stained with crystal violet. Plaques are visible as holes in the stained cell layer, one plaque is counted as one viral particle present in the diluted suspension.

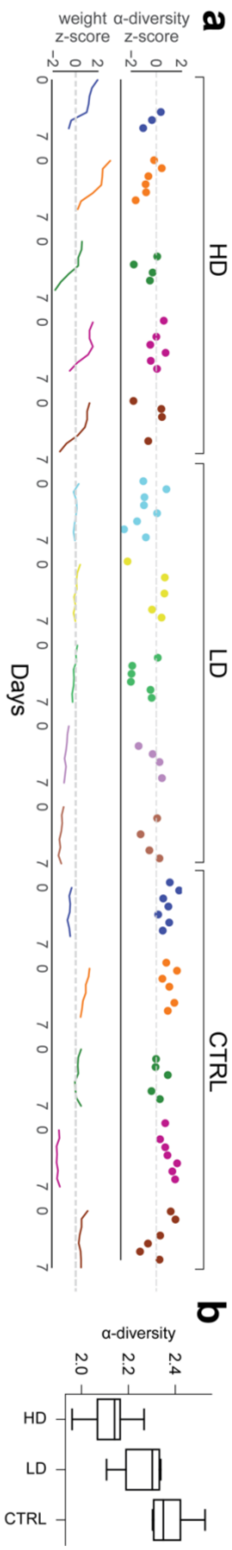

Supplementary Figure S2. Weight- and diversity loss during SARS-CoV-2 infection in mice. a z-scores of *α*-diversity (top) and body weight (bottom) over time; colors per mouse. **b** Difference in [g] of body weight from day of infection and six days after for the different experimental groups (HD: high viral dose, LD: low viral dose, CTRL: uninfected control).

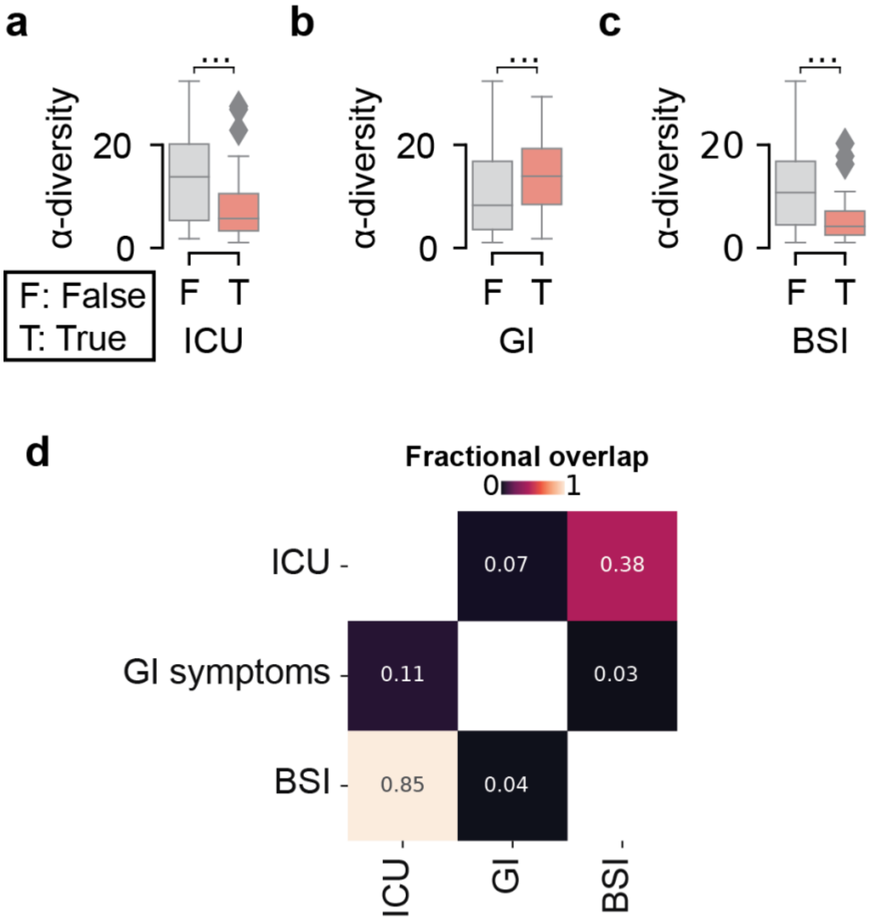

Supplementary Figure S3. Microbiome diversity is associated with clinical metadata. a-c *α*-diversity in patients with different clinical status; respectively, ICU treatment required, gastrointestinal symptoms reported, positive blood culture detected (***: 99%HDI <0, **: 95%HDI <0, BEST analysis of difference in means [24]). **d** Fractional overlap between clinical metadata categories in **a-c**.

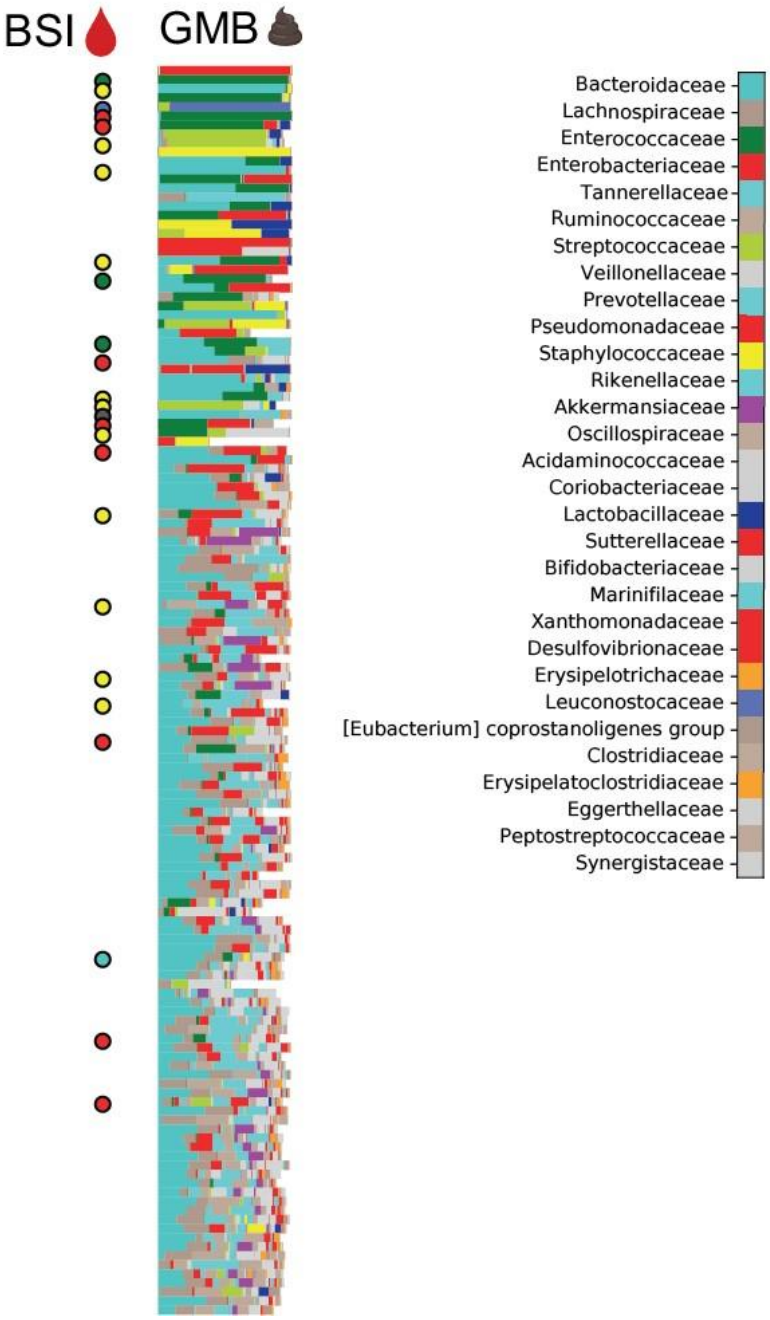

Supplementary Figure S4. Microbiome composition is associated with secondary bloodstream infections. All stool samples ordered by bacterial α-diversity (GMB) visualized as bars representing relative bacterial genus abundances, alongside microorganisms identified in the blood (BSI) using the same color scheme.

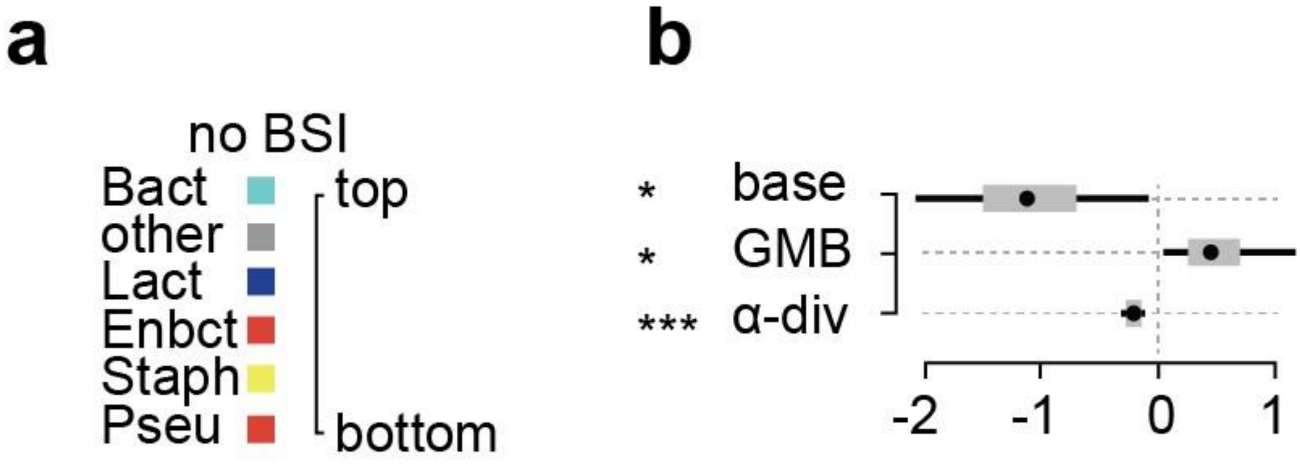

Supplementary Figure S5. Rank abundances in uninfected samples and global GMB association coefficients with BSIs. a Rank of BSI predictors in the GMB of uninfected samples. **b** Posterior association coefficient estimates of GMB predictors; ***: 99% HDI>0, **: 95%HDI>0, *: 90%HDI>0, .:85% HDI>0.

Supplementary Table S1: Disease Score for SARS-CoV-2 Infected Mice. Scoring system: Reduced activity/resistance = 1, if not moving = 2, ruffled fur=1, hunched posture = 1 or 2, heavy breathing =1

mouse ID	D0 (infection)	D1	D2	D3	D4	D5	D6	D7
CTRL_1	0	0	0	0	0	0	0	NA
CTRL_2	0	0	0	0	0	0	0	NA
CTRL_3	0	0	0	0	0	0	0	0
CTRL_4	0	0	0	0	0	0	0	0
CTRL_5	0	0	0	0	0	0	0	0
LD 1	0	0	0	0	0	0	0	0
LD 2	0	0	0	0	0	0	0	0
LD 3	0	0	0	0	0	0	0	0
LD 4	0	0	0	0	0	0	0	0
LD 5	0	0	0	0	0	0	0	0
HD 1	0	0	0	0	1	4	6	NA
HD 2	0	0	0	0	1	4	6	NA
HD 3	0	0	0	0	2	4	6	0
HD 4	0	0	0	0	1	4	6	0
HD 5	0	0	0	0	1	3	6	0

Supplementary Table S2: Clinical Characteristics of Patients with Confirmed COVID-19 at NYU Langone Health.

	N=65
Age (median, IQR)	51 (31-66)
**Sex**	
Male	58%
Female	42%
**Admission Type**	
Ambulatory	17%
Hospital	83%
**Comorbidities**	
Hypertension	38%
Hyperlipidemia	18%
Obesity	25%
Hypothyroid	5%
Diabetes Mellitus	32%
Chronic Viral Infection (HIV/Hepatitis)	10%
COPD	7%
ESRD/CKD	7%
Current Malignancy - blood	5%
Current Malignancy - other	2%
History of DVT	3%
Organ Transplantation	10%
SLE	3%
Immunosuppression	17%
IBD	22%
**Presenting Symptoms**	
Fever > 99F	46%
Cough	28%
Sputum	3%
Shortness of Breath	40%
Diarrhea	45%

**Hospital Course and Outcomes**	
ICU Admission	53%
Pneumonia	42%
Diarrhea	13%
Intubation	36%
Sepsis	23%
Encephalopathy	12%
Death	5%
Length of Stay (days, median, IQR)	37 (10-86)
**Chest Radiograph Findings**	
Bilateral haziness/opacities	38%
Unilateral haziness/opacities	3%
Bilateral Effusion	5%
Unilateral Effusion	3%
Consolidation	7%
Atelectasis	3%

Supplementary Table S3: Clinical Characteristics of Patients with Confirmed COVID-19 at Yale New Haven Hospital.

	N=37
Age (years)	62.52 ± 19.72 (n=33)
Sex (F | M)	39% | 61% (n=33)
BMI	31.22 ± 10.3 (n=25)
Race and Ethnicity	
Black or African-American	52% (n=17/33)
Hispanic or Latino	15% (n=5/33)
Asian	0% (n=0/33)
Caucasian	33% (n=11/33)
Other	0% (n=0/33)
Risk Factors	
None Admitted	16% (n=4/25)
Cancer within 1 year	4% (n=1/25)
Chronic Heart Disease	36% (n=9/25)
Hypertension	64% (n=16/25)
Chronic Lung Disease	20% (n=5/25)
Immunosupression	4% (n=1/25)
